# Lower‐Limb‐Predominant Axonal Sensorimotor Polyneuropathy With Severe Proximal Weakness and Possible Myopathic Involvement After Sleeve Gastrectomy: A Case Report of Delayed Recognition

**DOI:** 10.1002/ccr3.73813

**Published:** 2026-10-30

**Authors:** Altin Kuqo, Floreta Kurti, Erand Llanaj

**Affiliations:** ^1^ Department of Neuroscience Faculty of Medicine, University of Medicine Tirana Albania; ^2^ Neurology Service, University Hospital Center “Mother Teresa” Tirana Albania; ^3^ Department of Biomedical Sciences Faculty of Medical Technical Sciences, University of Medicine Tirana Albania; ^4^ Gastroenterology and Hepatology Service, University Hospital Center “Mother Teresa” Tirana Albania; ^5^ Department of General Practice and Family Medicine Medical School OWL, Bielefeld University Bielefeld Germany; ^6^ Translational Research and Health Services Research in General Practice, Medical School OWL, Bielefeld University Bielefeld Germany

**Keywords:** axonal polyneuropathy, bariatric surgery, diagnostic error, nerve conduction studies, sleeve gastrectomy

## Abstract

Bariatric surgery is increasingly performed in adolescents and young adults. Although sleeve gastrectomy preserves intestinal continuity and is often regarded as nutritionally low‐risk, it can still precipitate micronutrient deficiency, and obesity itself predisposes to thiamine deficiency, which can cause both Wernicke encephalopathy and a peripheral neuropathy. Peripheral neuromuscular complications after sleeve gastrectomy are less well characterized than those after malabsorptive procedures. An 18‐year‐old woman with class II obesity (body‐mass index 39.2 kg/m^2^) underwent laparoscopic sleeve gastrectomy and, about 10 weeks later, developed progressive lower‐limb weakness, distal paresthesiae, myalgia, cognitive fatigue, and loss of independent ambulation, with rapid weight loss (30 kg in three months) but no vomiting and no structured supplementation. Early nerve‐conduction studies were non‐diagnostic, and the presentation was attributed to a functional disorder, for which olanzapine was started; specialist reassessment was delayed until after objective progression. Repeat studies showed a lower‐limb‐predominant axonal sensorimotor polyneuropathy with preserved tibial and upper‐limb responses and no demyelinating features. The decisive finding was a discrepancy: Medical Research Council grade 1/5 proximal weakness out of proportion to the modest distal axonal loss, pointing to an unlocalized proximal process—lumbosacral radiculoplexus neuropathy or myopathy—for which needle electromyography of proximal and paraspinal muscles was the highest‐yield missing test. Vitamin B12, creatine kinase, and albumin were normal; thiamine, the wider micronutrient panel, and cerebrospinal fluid were never obtained. After rehabilitation, without documented micronutrient repletion, strength recovered to grade 3/5, and assisted walking returned. Nutritional deficiency was suspected but neither confirmed nor treated, so the etiology remains unresolved. The transferable lesson is diagnostic: severe neuromuscular injury can evolve within weeks of a restrictive procedure, nondiagnostic early tests do not exclude it, and objective signs should override a functional label.

## Key Clinical Message

After sleeve gastrectomy, progressive weakness, areflexia, and sensory loss demand objective reassessment for neuromuscular disease; nondiagnostic early tests do not exclude it, and a functional label becomes untenable once hard signs appear. Localize proximal weakness with needle electromyography and muscle imaging.

## 1 Introduction

Obesity is a chronic, multifactorial disease, and bariatric surgery is its most effective durable treatment when conservative measures fail. Operative volumes continue to rise, and sleeve gastrectomy is now the most commonly performed procedure in many regions [1]. Although it preserves intestinal continuity and carries a lower malabsorptive risk than gastric bypass, it can still precipitate micronutrient deficiency through reduced intake, vomiting, rapid weight loss, food intolerance, preexisting deficiency, and inadequate supplementation. Most surgical candidates have at least one micronutrient deficiency before the operation [2], and obesity itself predisposes to thiamine deficiency, so a normal or increased body habitus should not be taken to exclude it [3].

Neuromuscular complications after bariatric surgery span two categories [4, 5]. The first is Wernicke encephalopathy, a thiamine‐dependent emergency that is underrecognized and has been described after sleeve gastrectomy as well as bypass [6, 7]. The second is peripheral nerve and muscle disease, including length‐dependent axonal polyneuropathy and, less commonly, lumbosacral radiculoplexus neuropathy or myopathy [8, 9]. Thiamine deficiency can produce both; the presentation may be purely peripheral, yet the urgency derives from the central syndrome, whose injury may become permanent if untreated [10].

Thiamine reserves are small, sufficient for only two to three weeks, and can be exhausted within weeks of a restrictive procedure when intake is poor. In the largest single‐center series of vertical sleeve gastrectomy (147 patients), postoperative thiamine deficiency affected a substantial minority (of the order of one in six to one in five at 3–12 months, with postoperative nausea and vomiting among the predictors), and preoperative deficiency alone has been reported in roughly 2%–29% of candidates [11]. We report a young woman who developed a severe subacute lower‐limb sensorimotor syndrome with disproportionate proximal weakness and a probable myopathic component within weeks of sleeve gastrectomy, in whom specialist recognition was delayed for several months after an initial functional attribution and who partially improved after rehabilitation.

## 2 Case Presentation

An 18‐year‐old woman with class II obesity (preoperative weight 120 kg, height 175 cm, body‐mass index 39.2 kg/m^2^) underwent laparoscopic sleeve gastrectomy (Table 1). She had received no pharmacological obesity treatment; prior efforts were limited to unsupervised diets, with no completed supervised weight‐management program. No evaluation of the cause of obesity or of comorbidity was documented, and the only recorded preoperative assessment was the routine anesthetic review; operative details and any leak or stenosis evaluation were unavailable. Baseline micronutrient screening and a documented postoperative supplementation plan were likewise absent from the record; given that preoperative deficiency is common, their absence is an important gap.

**TABLE 1:** Clinical timeline and diagnostic inflection points.

Time from surgery/clinical phase	Symptoms	Objective findings	Interpretation	Inflection/missed opportunity
Preoperative	None reported	Class II obesity (BMI 39.2 kg/m^2^); no supervised programme	Nutritional vulnerability not characterized	Baseline micronutrient screen not documented
≈10 weeks after surgery	Progressive leg weakness, paresthesiae, myalgia, cognitive fatigue	Examination at onset not documented	Subacute postoperative neuromuscular syndrome	No micronutrient panel; no empiric thiamine
Early after symptom onset	Ongoing weakness	Initial NCS with limited needle EMG non‐diagnostic; brain MRI later reported normal; no spinal/plexus MRI	Early electrophysiology may be normal in evolving axonal injury; Brain MRI was reportedly normal but the formal report was unavailable; no dedicated spinal or lumbosacral‐plexus MRI was performed, so spinal cord, roots and plexus were not assessed.	Non‐diagnostic early tests over‐interpreted as excluding objective disease; no micronutrient panel; specialist review delayed ~7 months
Early after symptom onset	Distress	Not documented	Conversion disorder diagnosed; olanzapine started	Diagnostic anchoring: a functional/psychiatric attribution applied before the deficit evolved; an antipsychotic does not treat weakness, areflexia or an abnormal NCS and has no specific therapeutic role here
After progression, before specialist localisation was complete	Unable to walk unaided	MRC 1/5 proximal and distal; areflexia; L3–S2 sensory loss; proximal atrophy	Objective, progressive deficit incompatible with a purely functional attribution	Functional attribution required revision once fixed weakness, areflexia and abnormal electrophysiology emerged
At repeat electrodiagnostic assessment	As above	Repeat NCS: lower‐limb–predominant axonal sensorimotor polyneuropathy; preserved tibial; no conduction block across fibular head	Objective neuropathy confirmed; proximal deficit still unlocalised	Comprehensive needle EMG (paraspinals), CSF and plexus MRI not obtained to localize
Approximately 3 months after rehabilitation/follow‐up	Reduced disability; improving	MRC ~3/5; stands and walks with one aid	Partial recovery (MRC ~3/5; assisted walking) with rehabilitation, not documented repletion	—

Abbreviations: BMI, body mass index; CSF, cerebrospinal fluid; MRC, Medical Research Council; MRI, magnetic resonance imaging; NCS/EMG, nerve conduction studies/electromyography.

Approximately 10 weeks postoperatively she developed progressive, symmetric lower‐limb weakness with distal paresthesias and myalgia, advancing over weeks to an inability to walk independently, with cognitive fatigue and impaired concentration. There was no nausea, vomiting, or food intolerance; the nutritional vulnerability therefore rests on rapid weight loss (30 kg in the first three postoperative months) and the absence of a supplementation plan rather than on emesis. Oral and fluid intake, supplement adherence, and alcohol use were not separately documented; in the absence of biochemistry these would have been the strongest available proxy for an early nutritional cause. At initial assessment an early NCS with limited needle EMG was nondiagnostic, and the presentation was attributed to a functional (conversion) disorder, for which olanzapine was commenced. This attribution preceded full evolution of the deficit and any micronutrient testing; the fixed, severe weakness and areflexia described below were documented later, so the record does not establish that hard neurological signs were present and overlooked at that time.

The patient was not seen by a neurologist until approximately 7 months after onset, by which time the working diagnosis had remained functional or conversion disorder despite progression; the wording here is deliberately neutral, as the record does not permit attribution of the delay to any individual or team (Table 1). At maximal deficit, examination showed severe lower‐limb weakness (MRC grade 1/5 in hip flexion and extension, knee flexion and extension, and ankle dorsiflexion and plantarflexion), generalized lower‐limb areflexia, reduced tactile sensation in an L3–S2 distribution (stocking versus dermatomal pattern not characterized) and proximal muscle atrophy. Upper‐limb power and reflexes, ocular motility and cranial‐nerve testing, mental‐status examination, proprioception and vibration sense, and gait and truncal stability were not documented; ocular and mental‐status findings are needed to address the Wernicke spectrum, and proprioceptive findings to address dorsal‐column involvement (Table 2).

**TABLE 2:** Neurological examination and anatomical localization.

Domain	Finding	Localizing value
Motor (lower limb)	MRC 1/5: hip flexion/extension, knee flexion/extension, ankle dorsiflexion/plantarflexion	Severe weakness involving proximal and distal groups
Motor (upper limb)	Not documented	Defines extent; upper‐limb NCS normal
Sensory	Reduced tactile sensation, L3–S2 distribution; stocking versus dermatomal pattern not specified	Dermatomal/radicular pattern would support a radiculoplexus process; reduced SNAPs place the lesion at/distal to the dorsal root ganglion
Reflexes	Generalized lower‐limb areflexia	Peripheral nerve, motor‐axon, or root involvement
Cranial nerves/mental status	Ocular motility, nystagmus, and mental status not documented	Addresses the Wernicke spectrum
Proprioception/coordination	Vibration sense, proprioception, ataxia, and Romberg testing not documented	Addresses dorsal‐column involvement (B12, copper, vitamin E)
Functional status	Loss of independent ambulation	Disability driven mainly by the proximal deficit
Localization	Peripheral sensorimotor process; proximal weakness exceeds what distal NCS explain	Comprehensive needle EMG (incl. paraspinals) and plexus imaging needed; limited EMG and thigh MRI already suggest a myopathic component

## 3 Investigations

### 3.1 Neuroimaging

A brain MRI was reported as normal, although the formal radiology report was unavailable. Thigh‐muscle sequences demonstrated loss of muscle mass with fatty and fibrotic replacement—a pattern compatible with a myopathic or chronic neurogenic process—while pelvic and hip sequences reportedly showed no major abnormality; the formal report was pending, so these images require definitive interpretation (Figure S1). Because MRI of the Wernicke‐relevant regions (mammillary bodies and periaqueductal gray) can be normal even in active disease [10], a normal brain MRI does not exclude Wernicke encephalopathy. Dedicated spinal or lumbosacral plexus MRI was not performed, and a standard hip or thigh MRI does not substitute for dedicated plexus imaging.

### 3.2 Electrophysiology

Repeat NCS demonstrated a lower‐limb‐predominant axonal sensorimotor polyneuropathy (Table 3). Sensory studies showed reduced lower‐limb amplitudes (superficial peroneal and sural SNAPs) with normal to mildly slowed velocities and a preserved right median response, while motor studies showed markedly reduced peroneal CMAPs from extensor digitorum brevis bilaterally, with preserved right tibial and median CMAPs (Table 3). Because extensor digitorum brevis CMAPs can be low or absent in healthy individuals, the peroneal‐selective pattern is ideally confirmed against a more reliable distal muscle; an artifact is nonetheless less likely at this age, and the preserved tibial CMAP argues the abnormality is not solely technical.

**TABLE 3:** Nerve‐conduction studies: Measured values, derived metrics, and pattern adjudication.

Nerve (recording site)	Side/site	Latency/DML, ms	Amplitude	Reference^a^	CV, m/s^b^
**A. Measured values**					
Median SNAP (digit II)	R	2.2	38.8 μV	—	—
Superficial peroneal SNAP	L	2.1	3.6 μV	≥ 6 μV	48.8
Sural SNAP	R	4.0	6.8 μV	≥ 6 μV	30.0^c^
Sural SNAP	L	3.7	5.4 μV	≥ 6 μV	32.4
Median CMAP (APB)	R, wrist	2.6	12.4 mV	—	—
Median CMAP (APB)	R, elbow	6.7	12.4 mV	—	51.2
Tibial CMAP (abductor hallucis)	R, ankle	5.1	10.1 mV	≥ 4 mV	—
Peroneal CMAP (EDB)	R, ankle	5.9	1.6 mV	≥ 2 mV	—
Peroneal CMAP (EDB)	R, below fibular head	13.8	1.0 mV	—	38.2
Peroneal CMAP (EDB)	R, above fibular head	16.0	1.0 mV	—	45.0
Peroneal CMAP (EDB)	L, ankle	5.1	0.9 mV	≥ 2 mV	—
Peroneal CMAP (EDB)	L, below fibular head	12.2	0.6 mV	—	42.6
Peroneal CMAP (EDB)	L, above fibular head	13.7	0.6 mV	—	66.7^c^

Metric	Value	Interpretation
**B. Derived metrics**		
Right peroneal/tibial CMAP ratio^d^	0.16	Severe peroneal‐selective motor loss against a preserved right tibial response
Interside peroneal CMAP ratio (L/R, distal)	0.56 (≈44% reduction)	Both amplitudes at/below the lower limit of normal with temperature unrecorded → interpret cautiously; if confirmed, supports evaluation for a radiculoplexus or focal contribution
Upper‐ vs. lower‐limb gradient	Normal UL, abnormal LL	Length‐dependent topography

Question	Evidence and conclusion
**C. Pattern adjudication**	
Axonal vs. demyelinating	*Axonal*: Low amplitudes with only mild secondary slowing; DML not prolonged into the demyelinating range; peroneal velocity ≈87% of a representative lower limit of normal (not < 70%); no conduction block. An acquired demyelinating neuropathy is not supported
Focal fibular (peroneal) compression	*Unlikely as sole cause*: No conduction block across the fibular head and involvement is bilateral; a superimposed contribution cannot be fully excluded
Proximal weakness: radiculoplexus vs. myopathy vs. motor‐axonal	*Unresolved by NCS*: Routine NCS do not interrogate proximal muscle. The disproportionate proximal weakness and the interside amplitude difference are not explained by the modest distal axonal loss; a lumbosacral radiculoplexus neuropathy is at least as plausible as a nutritional myopathy. Reduced SNAPs place the lesion at/distal to the dorsal root ganglion, arguing against a pure radiculopathy. Needle EMG (including paraspinals), CK and CSF are required to discriminate
Chronicity	*Undetermined*: Cannot be graded from CMAP amplitudes; requires needle‐EMG reinnervation features. A “chronic‐stage” label should not be applied until then

Abbreviations: APB, abductor pollicis brevis; CK, creatine kinase; CMAP, compound muscle action potential; CSF, cerebrospinal fluid; CV, conduction velocity; DML, distal motor latency; EDB, extensor digitorum brevis; EMG, electromyography; LL, lower limb; LLN, lower limit of normal; NCS, nerve‐conduction study; SNAP, sensory nerve action potential; UL, upper limb.

^a^ Laboratory‐specific age‐ and height‐adjusted reference values were unavailable. Representative lower limits of normal are shown for context only and should not be interpreted as the performing laboratory's reference ranges: sural and superficial‐peroneal SNAP ≥ 6 μV; peroneal CMAP ≥ 2 mV; tibial CMAP ≥ 4 mV.

^b^ Amplitudes and conduction velocities are temperature‐dependent; limb temperature, F‐waves and H‐reflexes were not recorded.

^c^ Conduction velocities across short segments (fibular head) and those computed at very low amplitudes are unreliable and are shown only for completeness.

^d^ Ratios are exact arithmetic on measured amplitudes; no normative modeling is implied. A left peroneal/tibial ratio is not computed because the ipsilateral (left) tibial CMAP was not recorded, and cross‐side referencing would confound side with nerve.

Across the fibular head, peroneal amplitudes did not fall in a conduction‐block pattern, and there was no demyelinating‐range slowing. F‐wave and H‐reflex data, limb temperature, and laboratory‐specific reference values were unavailable; because cooling can reduce amplitudes and slow conduction, absolute and interside values must be read cautiously. Needle EMG was limited: the earlier study sampled left tibialis anterior (markedly reduced recruitment) and right vastus medialis (no recordable activation, partly limited by cooperation), interpreted as minimal myogenic involvement; the later study showed distal denervation with reinnervation in tibialis anterior, consistent with an axonal sensorimotor polyneuropathy of acute‐to‐subacute onset and chronic course. Comprehensive sampling of proximal and lumbosacral paraspinal muscles was not performed, and only the first of a reported nine pages was available, so the full quantitative dataset could not be verified. These limited findings leave the disproportionate proximal weakness incompletely localized between a radiculoplexus process, a myopathy, and severe motor‐axonal involvement.

### 3.3 Laboratory, Nutritional, and Cerebrospinal Fluid Assessment

The available biochemistry was limited but informative. Vitamin B12 was normal (462.8 pg/mL [341 pmol/L]; reference 191–663 pg/mL [141–489 pmol/L]), making B12 deficiency unlikely, and albumin was normal, arguing against advanced protein‐calorie malnutrition. Creatine kinase was low‐normal (17 U/L). Critically, serum thiamine (or erythrocyte transketolase activity), methylmalonic acid, copper, and ceruloplasmin, vitamin E, 25‐hydroxyvitamin D with calcium, phosphate, and parathyroid hormone, folate, zinc, prealbumin and a comprehensive metabolic panel were not obtained (Table 4), so the most time‐critical and treatable deficiencies were never measured.

**TABLE 4:** Nutritional, laboratory, and ancillary assessment: Missing and clinically indicated tests.

Test	Status	Why indicated	Interpretation caveat
Thiamine (whole‐blood/erythrocyte) or transketolase	Not obtained	Most time‐critical post‐bariatric deficiency; fits the phenotype	May be falsely normal after empiric repletion
Vitamin B12 normal; methylmalonic acid not obtained.	Normal (462.8 pg/mL [341 pmol/L]; ref. 191–663 pg/mL [141–489 pmol/L])	Neuropathy/myelopathy; cognitive change	B12 deficiency effectively excluded as a primary cause
Copper + ceruloplasmin	Not obtained	B12 mimic; myeloneuropathy	More typical of bypass; check with FBC
Vitamin E	Not obtained	Sensory ataxia, areflexia	Usually chronic
25‐OH vitamin D, Ca, PO_4_, PTH, ALP	Not obtained	Proximal weakness, myalgia (osteomalacic myopathy)	A candidate for a myopathic component
Creatine kinase	17 U/L (low‐normal)	Screens for myopathy	Low‐normal; argues against active necrotizing myopathy, not against chronic/non‐necrotizing
Needle EMG (incl. lumbosacral paraspinals, proximal muscles)	Performed (limited)	Distinguishes radiculoplexus vs. myopathy vs. motor‐axonal; stages chronicity	Performed but limited; comprehensive proximal/paraspinal sampling still the highest‐yield missing test
CSF (protein, cell count)	Not performed	Inflammatory radiculoplexus or GBS variant	Albuminocytological dissociation supports inflammation
Lumbosacral‐plexus/spinal MRI	Not performed	Radiculoplexus; exclude spinal cord disease	Use if radiculoplexus suspected
Brain MRI (FLAIR/DWI)	Performed; reported normal	Wernicke‐relevant regions	Formal report unavailable; normal MRI does not exclude Wernicke encephalopathy
Pelvic/hip/thigh MRI	Performed; formal report unavailable	Proximal weakness, atrophy, possible myopathy or chronic neurogenic process	Reportedly showed muscle‐volume loss with fatty/fibrotic replacement; requires definitive radiological interpretation
Albumin normal; folate, FBC, prealbumin and metabolic panel not obtained.	Not obtained	Anemia, malnutrition, repair capacity	—

Abbreviations: ALP, alkaline phosphatase; Ca, calcium; CSF, cerebrospinal fluid; DWI, diffusion‐weighted imaging; EMG, electromyography; FBC, full blood count; FLAIR, fluid‐attenuated inversion recovery; GBS, Guillain‐Barré syndrome; MRI, magnetic resonance imaging; PO_4_, phosphate; PTH, parathyroid hormone.

Cerebrospinal fluid analysis and antiganglioside antibodies were not tested; an elevated protein with normal cell count would have supported an inflammatory radiculoplexus neuropathy or Guillain‐Barré variant. A thiamine level obtained after empiric repletion may be falsely normal and should be interpreted accordingly.

## 4 Differential Diagnosis

Among nutritional causes, thiamine deficiency is the most time‐critical candidate given the postoperative onset, axonal pattern, areflexia, and cognitive symptoms; the framework in which early disease is thiamine‐related and later myeloneuropathy B12‐ or copper‐related is well described [7, 8]. The disproportionate proximal weakness merits specific scrutiny. A coexisting lumbosacral radiculoplexus neuropathy is a primary localization alternative, not a secondary aside: radiculoplexus and polyradiculoneuropathy phenotypes are recognized after bariatric surgery and rapid weight loss, are at times immune‐mediated, and would account for the proximal‐greater‐than‐distal weakness and interside asymmetry better than a purely length‐dependent process, which should be symmetric and distal‐predominant [9, 12]. The reduced sensory amplitudes place the lesion at or distal to the dorsal root ganglion and argue against a pure radiculopathy, which characteristically spares sensory responses, favoring a polyneuropathy or plexopathy over a root‐level process.

A myopathic component is positively, if incompletely, supported: limited needle EMG showed minimal myogenic change in vastus medialis, and the reported thigh‐muscle MRI showed muscle‐volume loss with fatty and fibrotic replacement. A low‐normal creatine kinase (17 U/L) argues against an active necrotizing myopathy but does not exclude a chronic, nutritional, or osteomalacic one (e.g., from vitamin D deficiency or protein‐calorie malnutrition); vitamin D, calcium, phosphate, and parathyroid hormone were not measured. B12 deficiency is effectively excluded by a normal serum B12 (462.8 pg/mL), and copper deficiency is less likely given the short interval and its stronger association with bypass, although it was not measured. An axonal Guillain‐Barré variant (acute motor and sensory axonal neuropathy) matters because it would change acute management; it is less likely given the subacute, surgery‐linked tempo and absent antecedent illness, but cannot be excluded without CSF, antiganglioside antibodies, and a complete electrodiagnostic timeline [13]. Chronic inflammatory demyelinating polyradiculoneuropathy is not supported, given the absence of demyelinating features or conduction block.

A peroneal compression neuropathy is suggested by the peroneal‐predominant motor findings and by rapid weight loss and immobility, but bilaterality and the absence of conduction block across the fibular head make isolated compression unlikely as the sole cause. Radiculopathy or spinal cord disease is addressed by the absence of a sensory level and would require spinal imaging. A purely functional attribution became untenable once the later objective signs—fixed MRC 1/5 weakness, generalized areflexia, and abnormal electrophysiology—were documented. Evidence for and against each diagnosis is summarized in Table 5.

**TABLE 5:** Differential diagnosis and evidence calibration.

Diagnosis	Supporting evidence	Contradicting/missing evidence	Likelihood
Thiamine (B1) deficiency/Wernicke spectrum	~10‐week onset; small stores; axonal sensorimotor pattern; areflexia; cognitive symptoms	No thiamine level; no vomiting; ocular/ataxia signs not documented; brain MRI reported normal (non‐excluding); recovery followed rehabilitation, not documented thiamine repletion	Leading safety diagnosis (unconfirmed)
Lumbosacral radiculoplexus neuropathy	Proximal>distal weakness; L3–S2 sensory pattern; interside asymmetry; recognized after rapid weight loss/bariatric surgery	No needle EMG (paraspinals), CSF, or plexus MRI; not yet evaluated	Primary localization alternative
Mixed micronutrient neuropathy (B12, copper, vitamin E)	Bariatric context; no documented surveillance; sensory + motor findings	B12 measured and normal (excludes B12 deficiency); copper, vitamin E, MMA not measured; copper less likely (more typical of bypass)	B12 excluded; others unassessed
Vitamin D/Ca/PO_4_ (osteomalacic myopathy)	Proximal weakness and myalgia; common after surgery and in obesity	Vitamin D/Ca/PO_4_ not measured; supported by vastus medialis myogenic EMG and thigh‐MRI fatty/fibrotic replacement; CK low‐normal	Probable co‐contributor (now partly supported)
Axonal GBS variant (AMSAN)	Areflexia; progressive weakness; axonal features	Subacute, surgery‐linked tempo; no antecedent illness; no CSF, antiganglioside antibodies, or full EMG timeline	Comparator; changes acute management; not excluded
AIDP/CIDP	Progressive; areflexia	No demyelinating features; no conduction block or temporal dispersion	Unlikely
Peroneal (fibular) compression neuropathy	Peroneal‐predominant CMAP reduction; weight loss/immobility increase susceptibility	Bilateral; no conduction block across fibular head; does not explain proximal weakness or broad sensory loss	Possible superimposed contributor
Radiculopathy/spinal cord disease	L3–S2 sensory pattern	Reduced SNAPs atypical for pure radiculopathy (spares SNAPs); no sensory level; no spinal MRI	Lower; exclude with spinal imaging
Functional attribution/conversion disorder	Initial non‐diagnostic imaging/NCS	Untenable after objective signs: fixed MRC 1/5 weakness, generalized areflexia, abnormal NCS	Excluded once objective signs present

*Note:* The likelihood column reflects a qualitative calibration against the available evidence rather than a quantitative probability: *Leading/primary* denotes a diagnosis positively supported by the phenotype yet unconfirmed for want of a discriminating test; *possible/plausible co‐contributor* denotes a diagnosis compatible with the context but not positively evidenced; *comparator* denotes a less likely diagnosis retained because it would change acute management; and *unlikely/excluded* denotes a diagnosis contradicted by an objective finding. No tier asserts a confirmed etiology.

Abbreviations: AIDP, acute inflammatory demyelinating polyradiculoneuropathy; AMSAN, acute motor and sensory axonal neuropathy; CIDP, chronic inflammatory demyelinating polyradiculoneuropathy; MMA, methylmalonic acid.

## 5 Treatment

No structured postoperative nutritional therapy or supplementation was documented, and there is no record of thiamine testing, parenteral thiamine, intravenous fluids, or glucose exposure during the diagnostic course. The case should therefore be presented not as treated post‐bariatric neuromuscular disease but as suspected nutritional or mixed disease with incomplete diagnostic and treatment documentation; the principal recorded intervention was physical rehabilitation. This absence of documented repletion is the single most important constraint on causal inference, because it removes any *ex juvantibus* argument. As general guidance—not a description of this patient's acute course—where thiamine deficiency is suspected in a malnourished or post‐bariatric patient, parenteral thiamine should be started empirically before any intravenous glucose (which can otherwise precipitate Wernicke encephalopathy) and without awaiting confirmation, with concurrent correction of magnesium, potassium, and phosphate [10, 14, 15].

## 6 Outcome and Follow‐Up

The patient underwent physical rehabilitation. At follow‐up approximately three months later there was meaningful improvement: lower‐limb strength recovered to around Medical Research Council grade 3/5, and she regained the ability to stand and walk with light support, using a single aid rather than two. This recovery is clinically valuable but is not ex juvantibus evidence for a nutritional etiology, because no specific thiamine or micronutrient repletion was documented; it can be attributed only to the observed period of rehabilitation, not to correction of a specific deficiency. This report is a retrospective reconstruction of a record the authors reviewed but did not generate: the diagnostic and therapeutic decisions, the investigations chosen, and the documentation were those of the original treating teams, and the missing biochemistry, comprehensive needle electromyography, and repletion data could not be obtained prospectively. The missing investigations are therefore reported as deficiencies of the available record rather than as analytical choices, and the inferences below are constrained accordingly.

## 7 Discussion

Sleeve gastrectomy, though it preserves intestinal continuity, is not nutritionally inert. Reduced gastric capacity, diminished intake, rapid weight loss, and any vomiting can deplete the small thiamine reserve within weeks, and postoperative thiamine deficiency has been reported in a substantial minority of patients after vertical sleeve gastrectomy specifically, not only after bypass [11]. Wernicke encephalopathy has likewise been documented after sleeve gastrectomy [6, 7]. Anchoring risk to procedure‐specific evidence, rather than extrapolating from malabsorptive operations, both strengthens the argument and avoids overstatement.

The phenotype and the literature must be reconciled carefully. The documented syndrome here is peripheral, whereas much of the bariatric thiamine literature concerns Wernicke encephalopathy, a central syndrome [5, 6, 7]. Thiamine deficiency can cause both a peripheral (“dry beriberi”) axonal neuropathy and Wernicke encephalopathy, so the peripheral presentation does not argue against a thiamine‐related process—but the central syndrome must be actively sought rather than assumed. The cognitive fatigue and impaired concentration warrant formal mental‐status and ocular‐motility examination, because the classic triad is usually incomplete and the diagnosis frequently missed [7], and because a normal brain MRI does not exclude Wernicke encephalopathy, which is a clinical diagnosis with frequently normal imaging [10]. Here the central syndrome was neither adequately characterized (no ocular‐motility or mental‐status documentation) nor excluded; the case therefore neither confirms nor excludes early Wernicke encephalopathy—the one diagnosis that is a true emergency.

Similarly, Ryan and Kinirons [16] described a Guillain‐Barré‐like neuropathy after esophagogastrostomy that progressed despite intravenous immunoglobulin and improved after correction of copper and vitamin D and vitamin E deficiencies, highlighting nutritional deficiency as a treatable mimic after gastric surgery.

The electrophysiology requires equally calibrated reading. The pattern is axonal, length‐dependent, and peroneal‐predominant, without demyelinating features. Preserved tibial and upper‐limb responses alongside markedly reduced peroneal CMAPs indicate a lower‐limb‐predominant, peroneal‐accentuated axonal process rather than a uniform severe neuropathy, and the absence of conduction block across the fibular head makes a purely demyelinating compressive fibular neuropathy unlikely.

The most informative observation is a discrepancy rather than a confirmation: routine distal NCS do not interrogate proximal muscle, and the MRC 1/5 proximal weakness is out of proportion to the modest distal axonal loss. The interside peroneal CMAPs also differ (left 0.9 mV versus right 1.6 mV; ratio 0.56, an approximately 44% interside reduction), but because both lie at or below the lower limit of normal and the recording temperature was undocumented, this may partly reflect technical factors. Taken with the proximal weakness, two processes are positively, if incompletely, supported and may coexist: a myopathic component (vastus medialis myogenic change on limited needle EMG, with reported thigh‐muscle fatty and fibrotic replacement) and a lumbosacral radiculoplexus neuropathy (proximal‐predominant weakness with interside asymmetry).

These differ in mechanism and management and should be evaluated directly with comprehensive needle EMG of proximal, distal, and lumbosacral paraspinal muscles (iliopsoas, quadriceps, adductors, gluteals, tibialis anterior, gastrocnemius), the formal thigh‐MRI report, creatine kinase, cerebrospinal fluid, and—where a radiculoplexus process is suspected—lumbosacral plexus or spinal MRI [9, 12]. Disease chronicity cannot be graded from CMAP amplitudes and should not be asserted without needle EMG reinnervation features.

Normal early investigations did not exclude evolving disease: axonal injury develops over time, and both MRI and an initial NCS/EMG may be normal early, so repeat electrophysiology—decisive here—should be pursued whenever symptoms progress. The contrast between the actual and the indicated diagnostic course is summarized in Figure 1. The initial functional attribution illustrates anchoring and premature closure; although objective neuromuscular disease is misdiagnosed as functional in only a minority of cases, the consequences can be serious [17], and here they were a delay in inexpensive, potentially curative treatment and exposure to an antipsychotic (olanzapine) with no role in this illness and with metabolic effects specifically counterproductive after metabolic surgery. Once objective signs—areflexia, fixed weakness, or abnormal electrophysiology—are present, the threshold for diagnostic revision should be low: any combination of progressive weakness, areflexia, and abnormal testing should prompt objective reassessment rather than continued observation.

**FIGURE 1:**
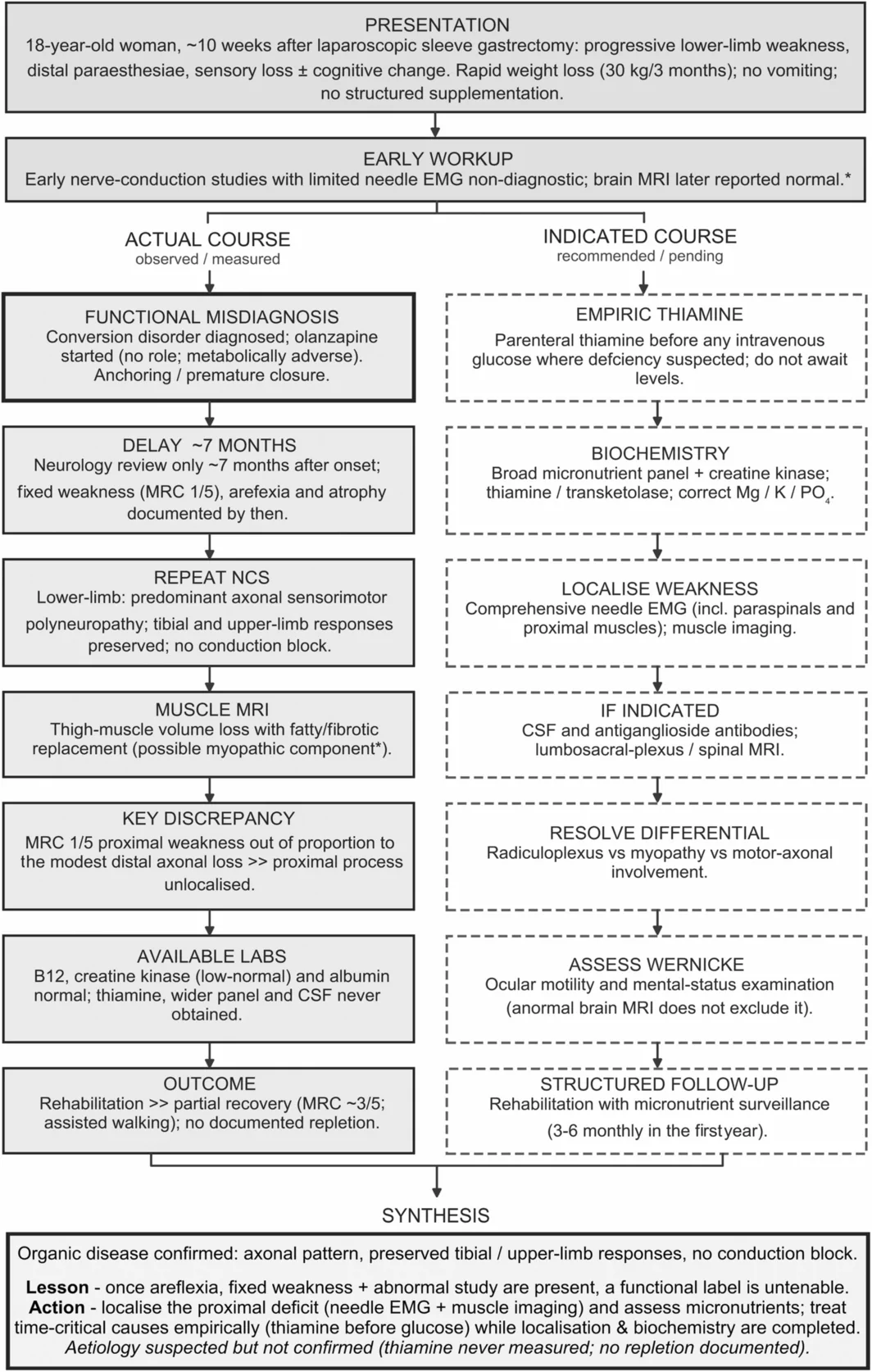
Diagnostic pathway for progressive weakness after bariatric surgery. Solid boxes indicate observed findings or interventions; dashed boxes indicate recommended diagnostic or therapeutic steps requiring confirmation. The pathway contrasts the observed clinical course (initial attribution to a functional disorder and treatment with olanzapine, specialist assessment delayed by approximately seven months and partial recovery with rehabilitation) with the recommended approach: Early empiric parenteral thiamine when deficiency is suspected, before intravenous glucose administration; correction of magnesium, potassium and phosphate deficiencies; comprehensive micronutrient testing and creatine kinase measurement; needle electromyography including proximal and paraspinal muscles, supplemented by muscle imaging; and cerebrospinal fluid analysis and spinal or plexus MRI when indicated. Available investigations included a reportedly normal brain MRI and thigh‐muscle MRI showing fatty or fibrotic replacement. Spinal and plexus MRI and cerebrospinal fluid analysis were not performed. *Formal radiology reports were unavailable for independent review. A normal brain MRI does not exclude Wernicke encephalopathy because of its limited diagnostic sensitivity. CK, creatine kinase; CSF, cerebrospinal fluid; EMG, electromyography; IV, intravenous; MRC, Medical Research Council; MRI, magnetic resonance imaging; NCS, nerve‐conduction study.

Two prevention points follow as general guidance, not a reconstruction of this late‐presenting patient's management.

First, in any malnourished or post‐bariatric patient with new neurological symptoms, empiric thiamine should precede intravenous glucose and not await confirmatory levels, with concurrent correction of magnesium, potassium, and phosphate [10, 14, 15].

Second, because most candidates are deficient before surgery and obesity itself predisposes to thiamine deficiency [3], preoperative screening, routine postoperative supplementation, and a structured surveillance schedule (three‐ to six‐monthly in the first postoperative year, annually thereafter, with symptom‐triggered testing sooner), reinforced by multidisciplinary follow‐up and a neurological red‐flag pathway, are the durable safeguards, especially in adolescents and young adults in whom adherence is harder to secure [2, 18, 19, 20].

### 7.1 What Cannot Be Concluded

Even with the information now available, a specific cause cannot be assigned. Thiamine and a broader neuropathy‐directed micronutrient panel, including copper, vitamin E, folate, and vitamin B6, were not measured, precluding attribution to a specific deficiency or recognition of combined deficiencies. Needle EMG was limited and did not sample proximal or paraspinal muscles; cerebrospinal fluid, antiganglioside antibodies, dedicated plexus and spinal imaging, and the formal radiology and full electrodiagnostic reports were not obtained. A normal vitamin B12 makes B12 deficiency unlikely, and a low‐normal creatine kinase argues against an active necrotizing myopathy, but neither identifies the mechanism.

The most defensible synthesis is a post‐bariatric axonal sensorimotor polyneuropathy with severe proximal weakness, a probable coexisting myopathic component, delayed recognition after an initial functional attribution, and a suspected but unconfirmed nutritional contribution; the partial recovery occurred with rehabilitation and without documented repletion and so does not establish causation. The principal limitations are the single‐patient retrospective design, the limited and partially reported electrodiagnostic data, the absence of dedicated spinal and plexus imaging, and confirmatory biochemistry, and a pending formal radiology report. The main strengths are the documented quantitative nerve‐conduction findings, the corroborating myogenic and muscle‐imaging signals, a documented functional outcome, and a transferable lesson about cognitive bias in a vulnerable demographic.

## 8 Conclusion

Severe neuromuscular disease can develop within weeks of sleeve gastrectomy and may be at least partly reversible; here strength improved to Medical Research Council grade 3/5 and assisted walking returned after rehabilitation. A practical red‐flag pathway, consistent with bariatric and Wernicke guidance, follows: in any post‐bariatric patient with new lower‐limb weakness, paresthesia, or cognitive change, characterize proximal weakness with needle electromyography (including proximal and paraspinal muscles) and muscle imaging, send a broad micronutrient panel with creatine kinase, give empiric parenteral thiamine before any intravenous glucose where deficiency is suspected, correct magnesium, potassium, and phosphate, and reserve a functional diagnosis until objective neuromuscular disease has been actively excluded [14, 15]. Once areflexia, fixed weakness, or an abnormal study is present, a functional label is untenable and should prompt objective reassessment. Comprehensive preoperative evaluation, routine postoperative supplementation, and a scheduled surveillance and neurological red‐flag pathway are the durable safeguards against irreversible injury, particularly in adolescents and young adults.

## Author Contributions

**Altin Kuqo:** conceptualization, investigation, data curation, writing – original draft, writing – review and editing. **Floreta Kurti:** investigation, data curation, writing – review and editing. **Erand Llanaj:** conceptualization, methodology, supervision, writing – review and editing, formal analysis, software.

## Funding

The authors have nothing to report.

## Disclosure

Patient Perspective: A patient perspective could not be obtained for this report.

## Ethics Statement

This report was prepared in accordance with the CARE (CAse REport) guidelines. The authors conducted a retrospective review of an existing clinical record and did not deliver the care described; the limitations in documentation reflect the source record and are stated explicitly throughout.

## Consent

Written informed consent was obtained from the patient for publication of this report and the associated data and figures, in accordance with the journal's patient consent policy. As the patient is 18 years of age, her own consent is sufficient. The text, tables, and figures contain no identifying information and the consent form is available to the Editor on request.

## Conflicts of Interest

The authors declare no conflicts of interest.

## Supporting information


**Figure S1:** Pelvic and thigh muscle volume loss with fatty infiltration on axial MRI. (A) Axial MRI at the pelvic/hip‐girdle level demonstrates marked bilateral reduction in muscle bulk involving the gluteal and proximal hip‐stabilizing musculature, including the gluteus maximus, gluteus medius, tensor fasciae latae, and sartorius muscles. (B) Axial MRI at the mid‐thigh level demonstrates generalized bilateral reduction in muscle bulk across the visualized thigh compartments, with increased intermuscular adipose tissue and intramuscular fatty infiltration. These findings support clinically relevant proximal muscle wasting and may help explain the severe proximal lower‐limb weakness. The imaging pattern is not etiologically specific and cannot, in isolation, distinguish nutritional myopathy, denervation, disuse‐related atrophy, or a mixed neuromuscular process.

## Data Availability

Due to the confidential nature of this case report and the risk of patient re‐identification, the underlying clinical data are not publicly available. Relevant de‐identified data may be made available by the corresponding author upon reasonable request, subject to applicable ethical, legal, and institutional requirements, and consistent with the patient's informed consent.
