# Supplementary material for: Lower‐Limb‐Predominant Axonal Sensorimotor Polyneuropathy With Severe Proximal Weakness and Possible Myopathic Involvement After Sleeve Gastrectomy: A Case Report of Delayed Recognition
Source: Clin Case Rep. 2026 Oct 30;14(11):e73813. doi: 10.1002/ccr3.73813 (PMC13721166; doi:10.1002/ccr3.73813)
Supplement: Supplementary file 1 [file CCR3-14-e73813-s001.pdf]

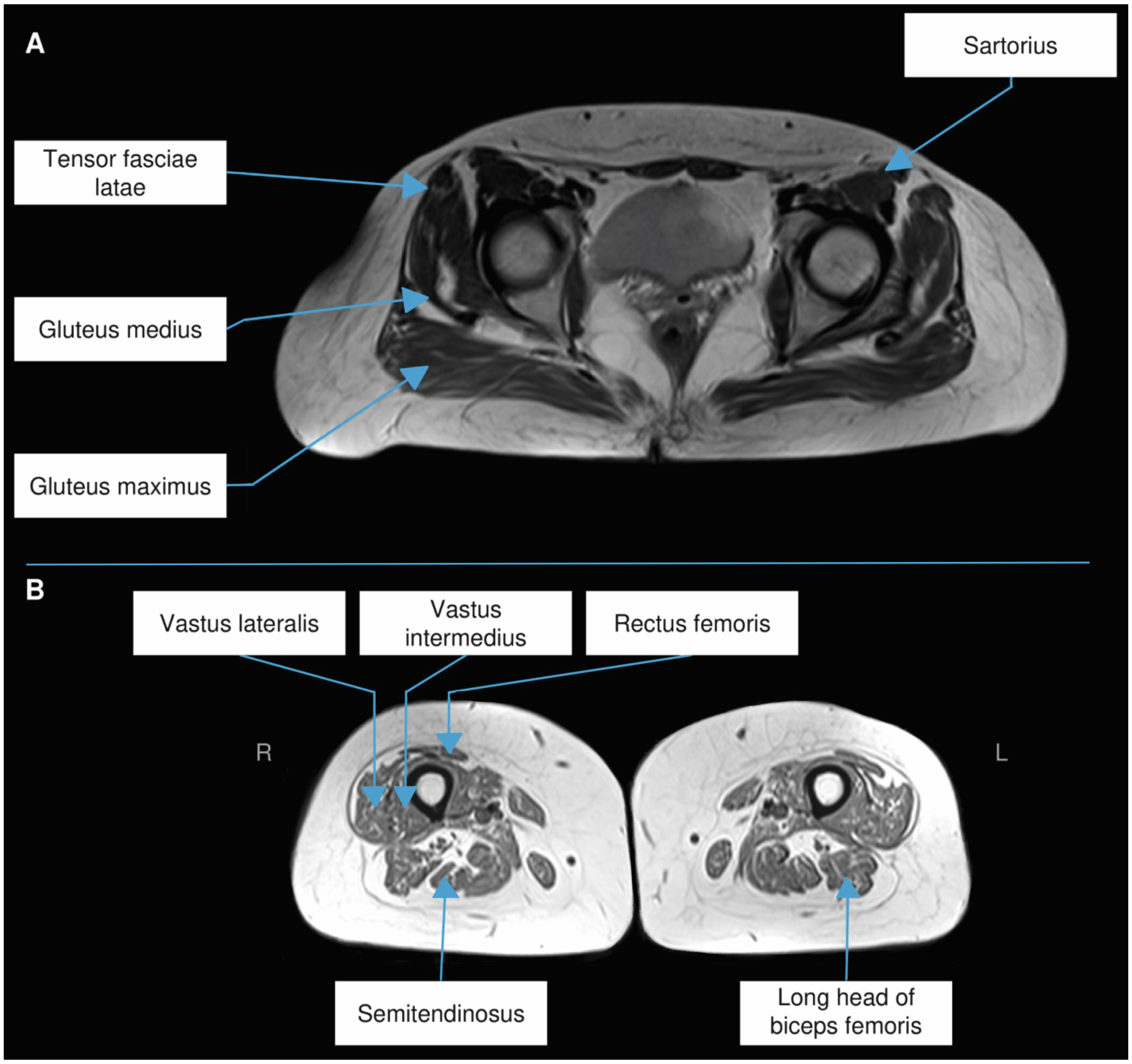

Supplementary Figure S1. Pelvic and thigh muscle volume loss with fatty infiltration on axial MRI. (A) Axial MRI at the pelvic/hip-girdle level demonstrates marked bilateral reduction in muscle bulk involving the gluteal and proximal hip-stabilizing musculature, including the gluteus maximus, gluteus medius, tensor fasciae latae and sartorius muscles. (B) Axial MRI at the mid-thigh level demonstrates generalized bilateral reduction in muscle bulk across the visualized thigh compartments, with increased intermuscular adipose tissue and intramuscular fatty infiltration. These findings support clinically relevant proximal muscle wasting and may help explain the severe proximal lower-limb weakness. The imaging pattern is not etiologically specific and cannot, in isolation, distinguish nutritional myopathy, denervation, disuse-related atrophy or a mixed
